# Organo-Nanocups Assist the Formation of Ultra-Small Palladium Nanoparticle Catalysts for Hydrogen Evolution Reaction

**DOI:** 10.3390/ma15072692

**Published:** 2022-04-06

**Authors:** Erik Biehler, Qui Quach, Clay Huff, Tarek M. Abdel-Fattah

**Affiliations:** Applied Research Center at Thomas Jefferson National Accelerator Facility and Department of Molecular Biology and Chemistry, Christopher Newport University, Newport News, VA 23606, USA; erik.biehler.16@cnu.edu (E.B.); qui.quach.13@cnu.edu (Q.Q.); clay.huff.12@cnu.edu (C.H.)

**Keywords:** palladium nanoparticles, organo-nanocups, catalysts, hydrogen evolution, hydride precursors

## Abstract

Ultra-small palladium nanoparticles were synthesized and applied as catalysts for a hydrogen evolution reaction. The palladium metal precursor was produced via beta-cyclodextrin as organo-nanocup (ONC) capping agent to produce ultra-small nanoparticles used in this study. The produced ~3 nm nanoparticle catalyst was then characterized via X-ray diffraction (XRD), transmission electron microscopy (TEM), ultraviolet-visible spectroscopy (UV-Vis), and Fourier transform infrared spectroscopy (FTIR) to confirm the successful synthesis of ~3 nm palladium nanoparticles. The nanoparticles’ catalytic ability was explored via the hydrolysis reaction of sodium borohydride. The palladium nanoparticle catalyst performed best at 303 K at a pH of 7 with 925 μmol of sodium borohydride having an H_2_ generation rate of 1.431 mL min^−1^ mL_cat_^−1^. The activation energy of the palladium catalyst was calculated to be 58.9 kJ/mol.

## 1. Introduction

Within the past 20 years, nanotechnology has allowed the development of smaller, more intricate systems and materials that have revolutionized many fields of study, including medicine, chemistry, and engineering. This has provided a great deal of positive change for numerous applications such as sensors, adsorbents, solar cells, and catalysis [1,2,3,4,5,6,7,8,9]. The unique properties of nanomaterials are ultimately driving their application in many fields, and their dimensions allow them to replace conventional materials to provide smaller, more efficient technologies [8,9,10]. This allows typically costly materials, such as precious metals, to be applied in economical ways with more efficient means, ultimately stretching global reserves further.

The transition metals, commonly known as precious metals or platinum group metals, have been prized for centuries due to their chemical resistivity caused by a full d-shell orbital. These metals are not only found in jewelry but also in many electrical and chemical applications due to their high conductivity and resistance to oxidation. Nanoparticles (NPs) of these precious metals, such as silver and gold, have shown impressive catalytic activity in hydrogenation and hydrogen evolution reactions [5,7,9,10,11,12]. Platinum and palladium NPs have also been used in a variety of organic reactions and have been studied in hydrogen evolution reactions [13,14,15,16,17]. Despite the high cost of these transition metals, nanoparticles of these elements are much less expensive than bulk and are more effective due to the innately high surface area to volume ratio of nanomaterials.

Hydrogen energy has been identified as a possible green alternative fuel source [18,19,20]. Due to the dangers and energy intensity of hydrogen gas storage, research has focused on producing the gas on an as-needed basis for fuel cells [21,22,23]. Sodium borohydride has been identified as a possible candidate for storing hydrogen gas for fuel cells due to its low density and impressive hydrogen content of 10.8% wt. However, the hydrolysis reaction of aqueous NaBH_4_ is slow and would require a catalyst to be effectively applied [21,24,25,26]. Gold and silver NPs have shown promise in the catalysis of this reaction in previous works, and palladium is known to be a strong catalyst for hydrogenation reactions from NaBH_4_ [5,7,9,10,14,15,16,27].

In this study, we utilized beta-cyclodextrin as an organo-nanocup capping agent to synthesize and form a network of ultra-small palladium nanoparticles (PdNPs). The PdNPs were characterized by various methods * applied in catalyzing the hydrogen generation reactions under various temperatures, pH, and reactant concentrations. At 303 K, the PdNPs catalyzed reaction produced a highest hydrogen amount at pH of 7 with 925 μmol of sodium borohydride.

## 2. Experimental

### 2.1. Materials

Palladium (II) chloride (Sigma-Aldrich, St. Louis, MO, USA, 99.9%), beta-cyclodextrin (Sigma-Aldrich, 99%), sodium borohydride (J.T. Baker, Phillipsburg, NJ, USA, 98%), deionized water (18 MΩ).

### 2.2. Synthesis

A 1 mM solution of Palladium (II) chloride was first created. A separate solution of aqueous beta-cyclodextrin (β-CD) with a concentration of 10 mM was also made. These solutions were combined in a ratio by volume of 1:6.4 then stirred for approximately 10 min. A 180 mM solution of sodium borohydride (NaBH_4_) was then made, chilled, and added to the above mixture in a 1:2 volume ratio. This mixture was then stirred for 120 min to aid in the formation of nanoparticle colloids (0.07 mM). All processes occurred at room temperature.

### 2.3. Characterization

The known identity of palladium nanoparticles was determined from the diffraction peaks seen using powder X-ray diffraction (XRD-Rigaku Miniflex II, Tokyo, Japan). The Cu Kα X-ray was emitted from copper target tube. The Cu Kβ radiation was filtered by nickel filters. The palladium nanoparticles were loaded on silicon wafer template and then scanned from 35° to 90°.

UV-Vis (Shimadzu UV-2600 UV-Vis Spectrophotometer, Kyoto, Japan) confirmed the successful reduction of Pd(II) to Pd(0).

Beta-cyclodextrin contains a major functional group that was identified using Fourier transform infrared spectroscopy (FTIR, Shimadzu IR-Tracer 100, Kyoto, Japan).

Transmission electron microscopy (TEM, JEM-2100F, JEOL Ltd., Akishima, Tokyo) helped characterize the nanoparticles produced by determining the dimensions of the nanoparticles and the electron diffraction patterns. We pipetted 1 µL of our catalyst onto a TEM grid and then dried it in an oven for 48 h prior to characterization.

### 2.4. Catalysis

Catalytic ability of our palladium nanoparticles was tested for evolution of hydrogen from aqueous sodium borohydride. The amount of hydrogen gas formed was quantified through the use of a gravimetric water displacement system [16,17]. The reactions of 625, 925, and 1225 µmol of aqueous NaBH_4_ in 100 mL of deionized water were catalyzed using 200 µL of the palladium nanoparticle colloids (0.07 M). The catalytic activity was compared in varied temperature (283 K, 288 K, 295 K, 303 K) as well as varied pH (6, 7, 8) by manipulation of the reaction chamber using an ice bath or heating mantle for temperature trials and hydrochloric acid or sodium hydroxide for pH trials. The contents of the reaction chamber were stirred throughout the 120-min trials to maintain equal distribution of the catalyst and reactant in the solution. Throughout the catalytic activity tests, the reactions were run with 925 µmol of reactant in 0.1 L of deionized water with a temperature of 295 K and a pH of 7 unless otherwise stated. The water displaced by the produced hydrogen gas was quantified with a Pioneer Balance (Pa124) made by Ohaus (Parsippany, NJ, USA) and using the SPDC Data Collector software (Ohaus, v2.01).

In the reusability test, 200 µL of palladium nanoparticles colloid was mixed with 100 mL of deionized water. Then 625 µmol of NaBH_4_ was added to the above mixture. The reaction was run at pH 7 and a temperature of 295 K. After the reaction no longer produced more hydrogen, the same amount of NaBH_4_ was added to test for the ability of palladium nanoparticles in continuing catalyzing the reactions.

## 3. Results and Discussion

### 3.1. Characterization

**Figure 1:**
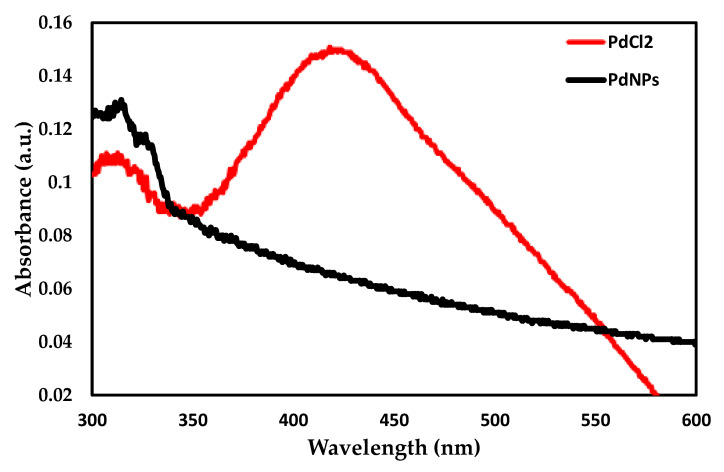
UV-Vis analysis of the spectra given off by PdNPs compared to that of PdCl_2_.

The UV-Vis Spectrum of PdNPs was displayed in Figure 1 and compared to that of PdCl_2_. The PdNPs showed absorbance peak at 315 nm, which was consistent with the absorbance range of previous studies [28,29]. The conversion of Pd(II) to Pd(0) was confirmed by the lack of the characteristic 415 nm peak [28,29,30]. These results confirmed the presence of PdNPs.

**Figure 2:**
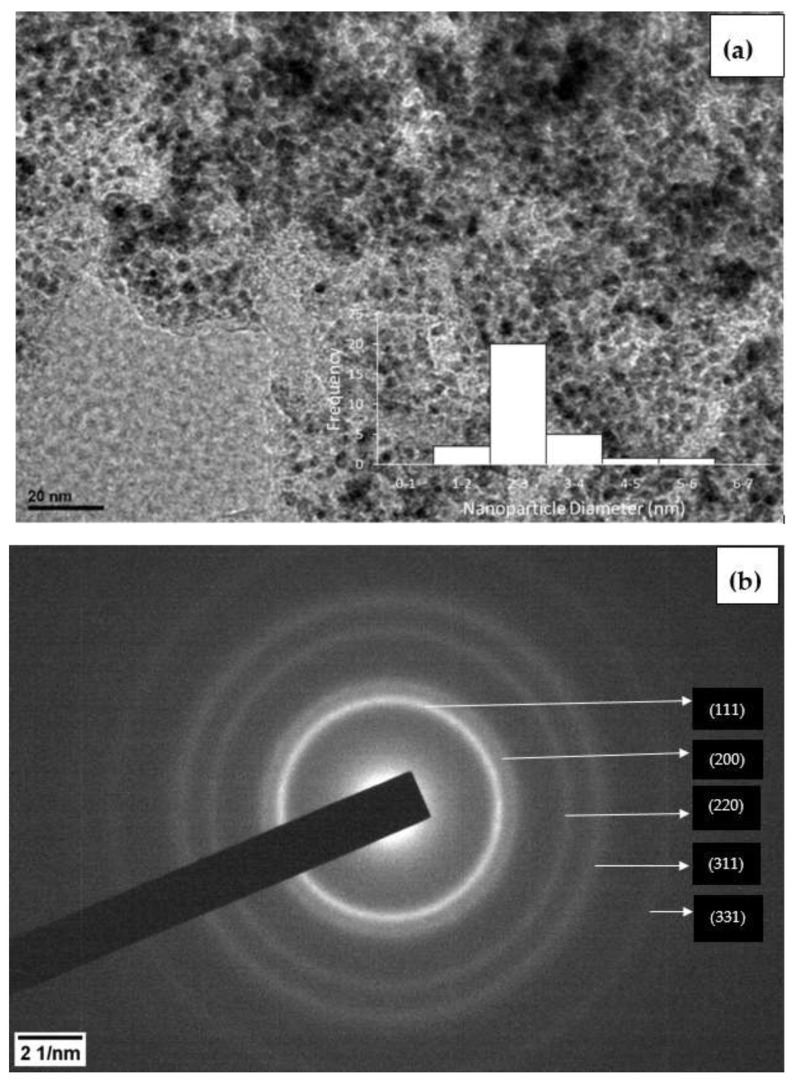

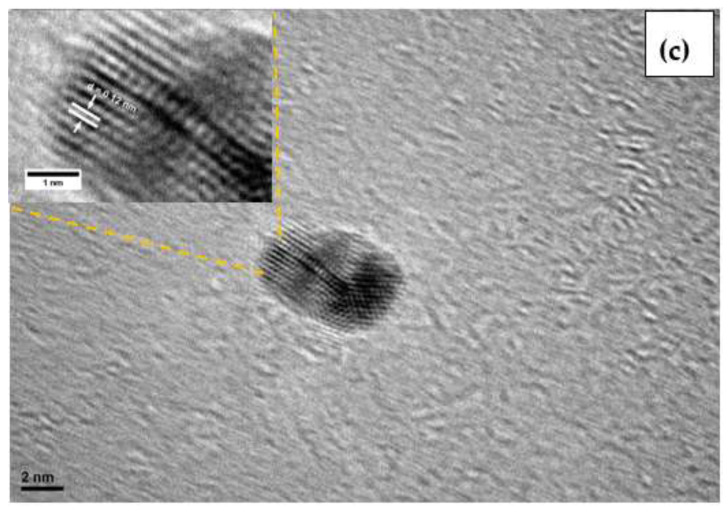
(**a**) Images of the palladium nanoparticles taken using TEM. The average diameter was determined to be 2.7 ± 0.8 nm. (**b**) The electron diffraction pattern of palladium nanoparticles. (**c**) Lattice fringes of palladium nanoparticles (d = 0.12 nm).

**Figure 3:**
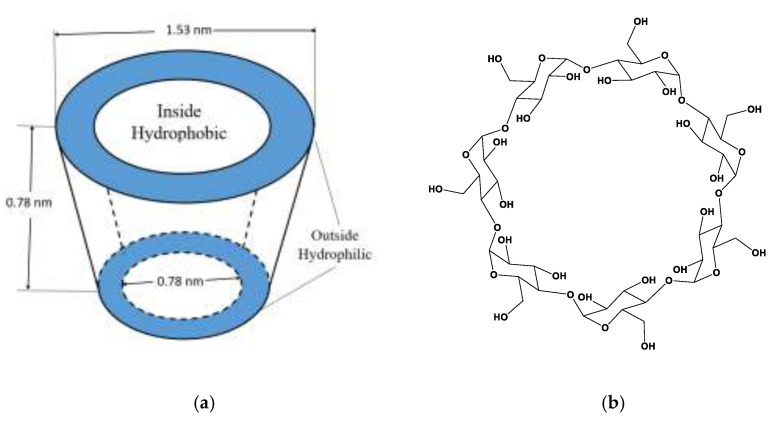
A graphical depiction of organo-nanocup capping agent of beta-cyclodextrin (**a**) and its molecular structure (**b**). Its shape consists of two hydroxyl rings, primary being the smaller of the two and secondary being the larger, and an internal cavity measuring 0.78 nm in diameter.

TEM micrographs (Figure 2) display the produced palladium nanoparticles (PdNPs). These images allowed the average diameter of our material to be determined to be 2.7 ± 0.8 nm. The TEM imaging again confirmed the successful synthesis of nanoparticles and showed that there was minimal agglomeration. Beta-cyclodextrin (Figure 3) as organo-nanocup (ONC) was used in conjunction with the nanoparticles to create a capping effect. The impressive uniformity of the produced nanoparticles combined with the facile nature of the synthesis makes beta-cyclodextrin an ideal capping agent to stabilized palladium nanoparticles.

Beta-cyclodextrin’s unique shape as nanocup aids in its efficiency as a capping agent [10]. The deep hydrophobic internal cavity in the center of the molecule confines nanoparticles within its narrow walls which prevents agglomeration by restricting their ability to grow unchecked [10]. This effect results in isolated and uniform particles, which are more efficient catalysts due to their increased available surface area. The structure of beta-cyclodextrin was depicted in Figure 3. The outer diameter, inner diameter and height of beta-cyclodextrin were 1.53 nm, 0.78 nm and 0.78 nm, respectively [10,31].

**Figure 4:**
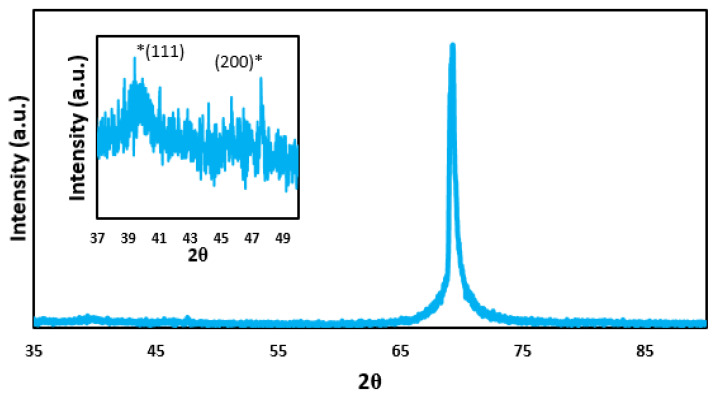
The characteristic peaks of the PdNPs on carbon tape from X-ray diffraction.

The XRD pattern (Figure 4) displayed the crystallinity of the palladium nanoparticles. The peaks at 40°, 48° were corresponded with the (111) and (200) planes for a face centered cubic (FCC) of palladium nanoparticles [28,29,30]. The highest peak at 69° was attributed to the silicon wafer in which the nanoparticles are loaded on [32]. That the other planes of the palladium nanoparticles were not detected could be due to the size of the nanoparticles. It was indicated in the literature that the nanoparticles with the average size below 5 nm will influence the XRD pattern [33]. TEM electron diffraction (Figure 2b) confirmed the other planes of (220), (311), and (331) of palladium nanoparticles [34,35]. Figure 2c shows the lattice fringe of palladium nanoparticles in which the d spacing is 0.12 nm.

**Figure 5:**
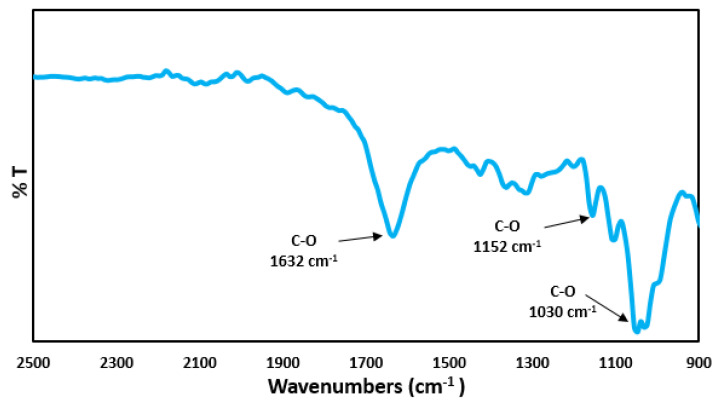
IR spectra of PdNPs.

Supported β-CD in PdNPs was confirmed by FTIR (Figure 5). The IR spectrum showed characterized peaks at 1030 cm^−1^, 1152 cm^−1^ and 1632 cm^−1^ that were corresponding with the vibrational stretching of glycosidic bond (C-O) of beta-cyclodextrin [36]. There was no strong peak at 2300 cm^−1^, which indicated that no NaBH_4_ remained in the nanoparticle colloids.

### 3.2. Concentration Effect on Catalytic Ability

**Figure 6:**
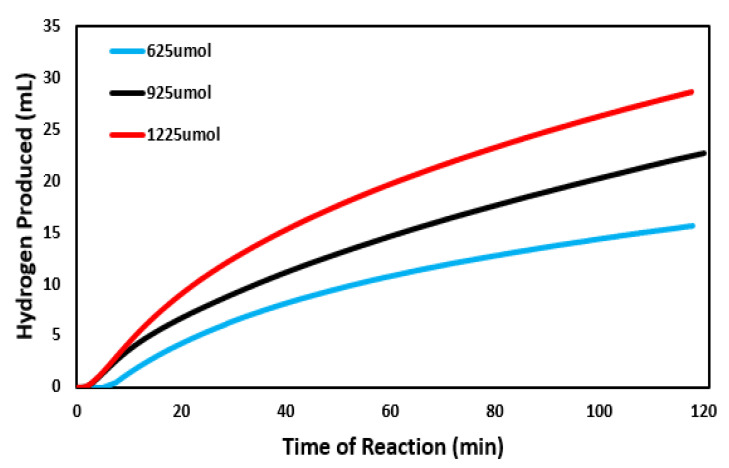
Volume of H_2_ generated versus time at varying sodium borohydride concentrations (625 µmol, 925 µmol, 1225 µmol). Reactions were run at ambient conditions for approximately 120 min.

Under varied concentrations of reactants, the PdNPs showed the greatest hydrogen production rate of 1.194 mL min^−1^ mL_cat_^−1^ with 1225 µmol of NaBH_4_ (Figure 6). The next best conditions for the nanoparticle catalyst was 925 µmol for the PdNPs (0.944 mL min^−1^ mL_cat_^−1^). The lowest concentration for hydrogen production rate was 635 µmol of NaBH_4_ producing H_2_ gas at a rate of 0.650 mL min^−1^ mL_cat_^−1^. Based on Figure 6 it is clear that there is a direct relationship between the concentration of NaBH_4_ used in the reaction and the amount of hydrogen produced. This agrees with Le Chatelier’s principle and Equation (1), where an increase in the reactants results in a shift to the right, increasing the products.

BH_4_^−^ + 2H_2_O → BO_2_^−^ + 4H_2_ (1)

### 3.3. Effect of pH on Catalytic Ability

**Figure 7:**
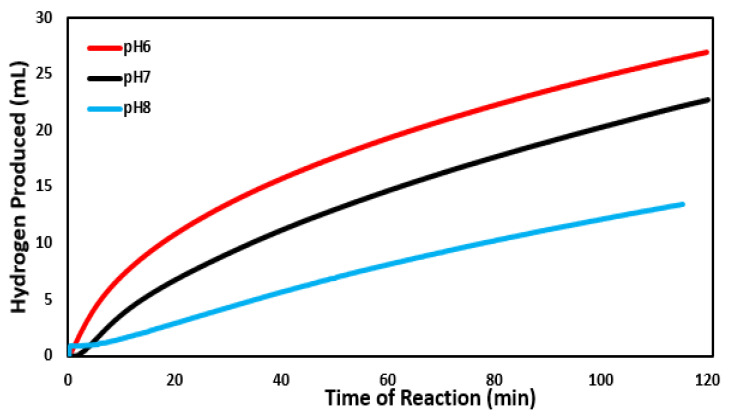
The amount of hydrogen gas produced in mL versus time in minutes as catalyzed by the palladium nanoparticles under varied solution pH’s (pH 6, 7, 8). The reactions were run at 295 K with 925 µmol of NaBH_4_ for roughly 120 min.

When the reaction was run using solutions of different pH’s, the highest rate hydrogen production rate was seen at acidic conditions (pH 6) with a rate of 1.121 mL min^−1^ mL_cat_^−1^ (Figure 7). This was then followed by pH 7 and pH 8 with evolution rates of 0.944 mL min^−1^ mL_cat_^−1^ and 0.560 mL min^−1^ mL_cat_^−1^, respectively. It is known that the formation of borate ions occurs at lower pH, which increases the reaction rate of the uncatalyzed hydrolysis reaction (Equation (1)) [23].

### 3.4. Activation Energy and Effect of Temperature on Catalytic Ability

**Figure 8:**
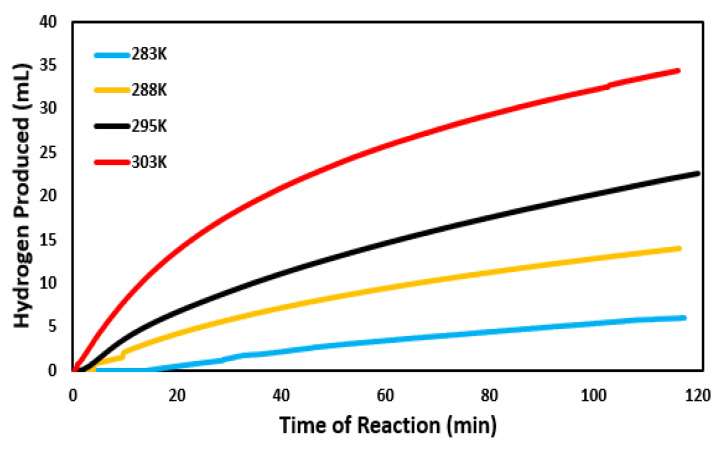
The varied amount of hydrogen produced by the hydrolysis of NaBH_4_ versus time as catalyzed by the PdNPs at 283 K, 288 K, 295 K, and 303 K. The reactions conditions included pH 7 and a concentration of 925 µmol of NaBH_4_ for upwards of 120 min.

**Figure 9:**
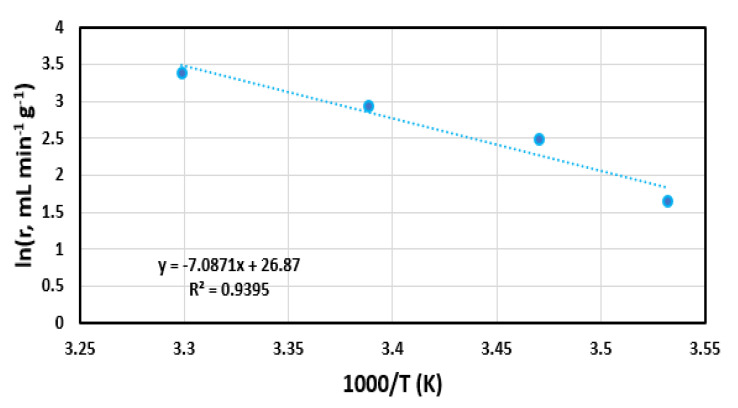
Arrhenius Plot created from the temperature data allowed for the calculation of the activation energy of the NaBH_4_ reaction catalyzed by the PdNPs.

Under varied temperatures, palladium nanoparticles produced the most hydrogen at 303 K with a rate of 1.431 mL min^−1^ mL_cat_^−1^ (Figure 8). For the 283 K and 288 K temperature conditions, rates of 0.255 mL min^−1^ mL_cat_^−1^ and 0.585 mL min^−1^ mL_cat_^−1^ were observed, respectively. Finally, a hydrogen generation rate of 0.944 mL min^−1^ mL_cat_^−1^ was seen at 295 K (Figure 8). Based on Figure 8 and Equation (2), the activation energy was calculated from the temperature trials to be 58.9 kJ/mol (Figure 9). Palladium nanoparticles displayed a similar activation energy when compared to other catalysts in literature (Table 1). The activation energy of Pd nanoparticles in this study is very attractive compared to most of the catalysts reported in the literature. In this study, it was conducted in ambient condition in the range of 273 K to 303 K.

lnK = lnA − E_a_/RT (2)

**Figure 10:**
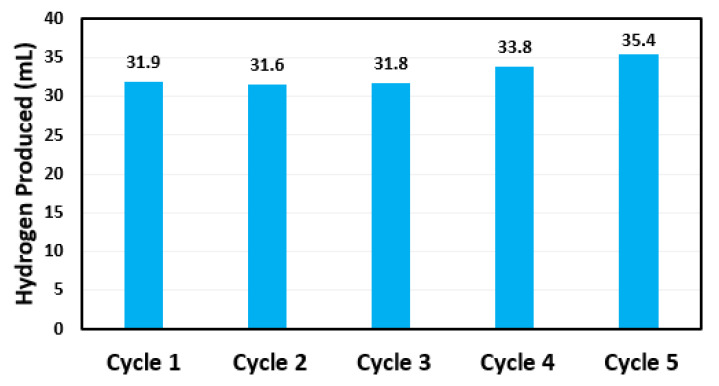
Reusability of the same PdNPs material for the reaction of sodium borohydride in water. Each trial ran for 120 min at 295 K and used 925 μm of NaBH_4._

Figure 10 depicts the stability of our PdNPs catalyst over the course of five consecutive hydrogen generation trials. Each trial produced similar volumes of hydrogen with an average volume of 32.9 mL observed among all trials. These results show a clear benefit to our catalyst as it can be utilized multiple times without a significant change in productivity.

**Scheme 1:**
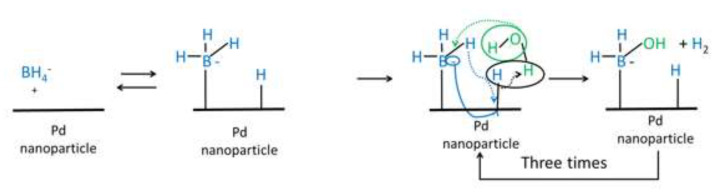
Our proposal for the mechanism of how sodium borohydride is hydrolyzed by catalysts from the group ten metal nanoparticles.

Table 1 is based on the Michaelis–Menten model for metal catalyzed hydrolysis of aqueous NaBH_4_ (Equations (3) and (4)) [9,11,12,17]. The proposed mechanism and its equations show how both the active catalytic metal site and an adjacent unoccupied site can stabilize the borohydride complex with the metal and the hydride ions. This indicates that the surface area of a catalyst is incredibly important to this reaction as unoccupied metal sites are also shown to aid in catalysis. The Scheme 1 also depicts the multi-electron process of this reaction, in which the last step repeats until tetrahydroxyborate is released from the metal site. Therefore, it is believed that a successful catalyst facilitates the movement of electrons between adjacent sites.

M + BH_4_^−^ ↔ MH + MBH_3_^−^ (3)

MH + MBH_3_^−^ + 4H_2_O → M + 4H_2_ + [B(OH)_4_]^−^ (4)

## 4. Conclusions

The ultra-small palladium nanoparticles were produced with the assistance of beta-cyclodextrin as organo-nanocup capping agent. The nanoparticles were then characterized via UV-Vis, TEM, FTIR, and XRD before application as a catalyst in the hydrolysis of sodium borohydride. The resultant ~3 nm Pd nanoparticles performed well as a catalyst with great usability, with an activation energy calculated to be 58.9 kJ/mol. The palladium nanoparticles show promise as a catalyst for application in sodium borohydride-based fuel cells.

## Figures and Tables

**Table 1:** Reported activation energies for NaBH_4_ hydrolysis by catalyst.

Catalyst	E_a_ (kJ/mol)	T (K)	Reference
AuNPs	54.7	283-303	[10]
Au/MWCNTs	21.1	273–303	[11]
Ag/MWCNTs	44.5	273–303	[12]
Pd/C	28.0	283–323	[16]
Pt-Pd-CNTs	19.0	302–332	[17]
Ru nanoclusters	41.0	298–318	[37]
Ni nanoclusters	54.0	298–318	[38]
Ni-Ru nanoclusters	52.7	288–348	[39]
Co/Fe_3_O_4_@C	49.2	288–328	[40]
Co–Cr–B	44.5	293–333	[41]
Cu-Fe-B	57	285–333	[42]
CuNWs	42.5	298–333	[43]
Cu based catalyst	61.2	293–313	[44]
PtMWCNT	46.2	283–303	[45]
PtNPs	39.2	283–303	[46]
AgNPs	50.3	273–303	[47]
PdNPs	58.9	273–303	This Work

## Data Availability

The data presented in this study are available on request from the corresponding author.
